# Magnetic field strength dependent SNR gain at the center of a spherical phantom and up to 11.7T

**DOI:** 10.1002/mrm.29391

**Published:** 2022-07-18

**Authors:** Caroline Le Ster, Andrea Grant, Pierre‐François Van de Moortele, Alejandro Monreal‐Madrigal, Gregor Adriany, Alexandre Vignaud, Franck Mauconduit, Cécile Rabrait‐Lerman, Benedikt A. Poser, Kâmil Uğurbil, Nicolas Boulant

**Affiliations:** ^1^ University of Paris‐Saclay, CEA, CNRS, BAOBAB NeuroSpin Gif sur Yvette France; ^2^ Center for Magnetic Resonance Research University of Minnesota Minneapolis Minnesota USA; ^3^ Faculty of Psychology and Neuroscience Maastricht University Maastricht The Netherlands

**Keywords:** field strength, signal‐to‐noise ratio, volume coil

## Abstract

**Purpose:**

The SNR at the center of a spherical phantom of known electrical properties was measured in quasi‐identical experimental conditions as a function of magnetic field strength between 3 T and 11.7 T.

**Methods:**

The SNR was measured at the center of a spherical water saline phantom with a gradient‐recalled echo sequence. Measurements were performed at NeuroSpin at 3, 7, and 11.7 T. The phantom was then shipped to Maastricht University and then to the University of Minnesota for additional data points at 7, 9.4, and 10.5 T. Experiments were carried out with the exact same type of birdcage volume coil (except at 3 T, where a similar coil was used) to attempt at isolating the evolution of SNR with field strength alone. Phantom electrical properties were characterized over the corresponding frequency range.

**Results:**

Electrical properties were found to barely vary over the frequency range. Removing the influence of the flip‐angle excitation inhomogeneity was crucial, as expected. After such correction, measurements revealed a gain of SNR growing as B_0_^1.94 ± 0.16^ compared with B_0_^2.13^ according to ultimate intrinsic SNR theory.

**Conclusions:**

By using quasi‐identical experimental setups (RF volume coil, phantom, electrical properties, and protocol), this work reports experimental data between 3 T and 11.7 T, enabling the comparison with SNR theories in which conductivity and permittivity can be assumed to be constant with respect to field strength. According to ultimate SNR theory, these results can be reasonably extrapolated to the performance of receive arrays with greater than about 32 elements for central SNR in the same spherical phantom.

## Abstract 2

Click here for author‐reader discussions

## 1 INTRODUCTION

Gains in SNR and contrast‐to‐noise ratios have fueled ongoing efforts for slow but steadily increasing magnetic fields in MRI.^1^, ^2^, ^3^, ^4^, ^5^, ^6^, ^7^, ^8^ In this context, the highest field encountered for human MRI to date is 10.5 T at the University of Minnesota (CMRR) when it was first brought up to its nominal field strength in 2014 and first human images reported in 2020.^9^ A few years later, first images were acquired at CEA at 11.7 T^10^ with a whole‐body magnet,^11^ while first in vivo images are expected in 2022–2023. The benefits of the research and development accompanying those efforts are unquestionable, as it has produced many new technologies of interest for the entire MRI field. Exploiting these extremely high magnetic fields for biomedical research, however, requires understanding and quantifying the SNR and application‐specific contrast‐to‐noise ratio gains expected with increasing field strength.

In parallel to the interest in such high field instruments, calculations and electromagnetic simulations of ultimate intrinsic SNR (uiSNR) have been developed over the years (12, 13, 14, 15, 16, 17, 18) to determine not only the achievable gains with B_0_ but also the available room for improved performance of current RF coils.^19^ Aside from the receive sensitivity contribution, the gain of signal with B_0_^2^ can be ubiquitously found in the literature from the high‐temperature approximation of the spin polarization combined with Faraday's law; however, the theoretical assessment of the noise is more complex. In a seminal paper, Hoult derived an analytical expression quantifying the SNR in a spherical phantom of known conductivity, and for an idealized spherical RF probe producing a circularly polarized (CP) field at the center,^20^ when the coil was lossless and only noise coming from the sample was considered. The RMS voltage noise was found to increase linearly at low fields and sublinearly at higher fields, hence overall returning a supra‐linear gain of SNR with B_0_ resulting from complex E‐field interferences when integrating over the volume. More practically, Pohmann et al^21^ obtained measurements with state‐of‐the art multireceive arrays in the human brain at 3, 7, and 9.4 T, reporting a B_0_^1.65^ dependence of SNR over the cerebrum after correcting for the inhomogeneity of the RF excitation and the change of MR parameters (T_1_, T_2_*). Strictly speaking, however, in this effort it is impossible to disentangle the impact of the field strength alone from the one arising from the RF coils, including receive sensitivity; although the RF coils used all had 32 receive channels, they were not necessarily similar in the coil layout nor the associated electronics. The result naturally is of great practical value but hard to compare with theory because of the coil diversity and the change of electrical properties, along with their uncertainties, of the biological tissues with respect to field strength.

As a result, it remains of great academic and practical interest to assess the SNR contribution from the B_0_ field alone, with other parameters fixed. We therefore report in this study the measurement of SNR at the center of a spherical phantom across three sites covering field strengths of 3 T, 7 T, 9.4 T, 10.5 T, and 11.7 T with nearly identical experimental setups and conditions, and we compare the results with uiSNR theory.

## 2 METHODS

### 2.1 Radiofrequency coils

**FIGURE 1:**
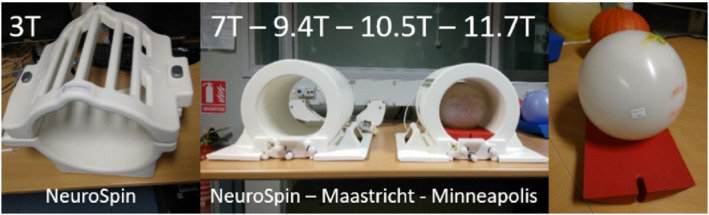
Photographs of the RF volume coils and phantom used for the measurements. The 7T, 9.4T, 10.5T, and 11.7T coils had strictly the same design and dimensions. The same exact phantom was shipped from one site to the next to keep experimental conditions as identical as possible

All utrahigh‐field 7T, 9.4T, 10.5T, and 11.7T experiments used identical 16‐rung shielded birdcage tune‐up service coils (QED, Mayfield Village, Ohio, USA) tuned and matched at their respective nominal frequencies and available at all participating sites. Figure 1 provides a view of the different coils and the same phantom and holder used throughout all experiments. The inner diameter of the utrahigh‐field coils was 215 mm with an end‐ring to end‐ring length of 229 mm. A shield with an inner diameter of 233 mm and length of 268.5 mm aided overall stability and reduced radiation. All utrahigh‐field tune‐up coils had four identical feed points and were driven with fixed transmit phases of 0, 90, 180, and 270. For the 3T experiment, because the same coil design was not available, we used a standard 16‐rung unshielded head birdcage (QED) with an inner diameter of 270 mm and a length of 310 mm. The unshielded 3T birdcage coil is closer to an optimal setup at 3 T than the shielded case at higher fields.^22^, ^23^ Noise figures (NFs) of the preamplifiers were also provided by the manufacturer of the coils and were 0.5, 0.8, 0.8, 0.77 and 0.8 dB at 3 T, 7 T, 9.4 T, 10.5 T and 11.7 T, respectively.

### 2.2 Magnetic resonance scanners

Measurements were performed at 3 T (NeuroSpin), 7 T (NeuroSpin; Maastricht University, CMRR), 9.4 T (Maastricht University), 10.5 T (CMRR), and 11.7 T (NeuroSpin). All experiments were carried out on Siemens platforms (Siemens Healthcare, Erlangen, Germany) using VE12 (3 T, 7 T, 10.5 T, and 11.7 T) and VB17 (7 T, 9.4 T) software and associated hardware. The reader should note that the usual terminology with rounded numbers for field strength is used in this work. The more exact values based on Larmor frequency (ie, 2.89 T, 6.98 T, 9.39 T, 10.5T, and 11.73 T) were otherwise used in all fitting procedures.

### 2.3 Phantom

The phantom consisted of a 16.5‐cm inner diameter sphere filled with saline water (4.6 g/L NaCl and 10 g/L agar). After measurements at 3 T, 7 T, and 11.7 T at NeuroSpin, the phantom was shipped to Maastricht for scans at 7 T and 9.4 T, continuing its journey for final measurements at 7 T and 10.5 T at the CMRR. The same holder positioning the phantom approximately at the center of the coil was used to eliminate any phantom‐related variation in SNR results. Because electrical properties can be sensitive to temperature, the phantom was placed in the scanner room for several days to equilibrate with the room temperature, controlled to be within the 18–20°C range, before scanning. The relative permittivity and conductivity were measured over the 128–500‐MHz range using the EpsiMu (Multiwave Technologies SAS, Marseille, France) and DAK‐12 (Speag, Zürich, Switzerland) technologies at NeuroSpin and CMRR, respectively. Both measurements agreed well and revealed only a few percent variations over the two sites, yielding on average for conductivity (σ) 0.98, 1.00, 1.01, 1.01 and 0.99 S/m and for relative permittivity (ε_r_) 76.7, 76.3, 76.2, 75.7, 75.9 at 3 T, 7 T, 9.4 T, 10.5 T, and 11.7 T, respectively. To a good approximation, the values were thus found to be constant throughout the 128–500‐MHz range. Accuracy of 3%–5% was provided for these measurements by the manufacturers of the electrical properties measuring devices.

### 2.4 Signal‐to‐noise ratio measurements

The same measurement protocol was repeated for each different field strength and site. A 3D gradient‐recalled echo (GRE) sequence was used (coronal orientation, Repetition time (TR) = 30 ms, TE = 3 ms, res = 1.5 × 1.5 × 1.5 mm^3^, Field of View (FOV) = 192 × 192 × 192 mm^3^, matrix = 128 × 128 × 128, bandwidth per pixel = 400 Hz) at several flip angles and TEs to recover the T_1_ and T_2_* value from fitting the signal with the theoretical signal equation. The B_1_^+^ field per volt was measured in each scenario by using the actual flip angle imaging (AFI) sequence^24^ to deduce the true flip angles to be used in the T_1_ fitting procedure and correct for the transverse magnetization produced at the center of the phantom. The voltages of the RF pulses were tuned in order to obtain in the center a flip angle within the range of 60°–80° at all field strengths and maintain good sensitivity in the AFI sequence. The raw data were exported and the complex images were reconstructed offline by Fourier transform. The process was repeated with an RF voltage of 0 V to calculate the noise contribution by taking the SD of the real part of the voxel values. The signal was estimated by averaging the intensities of the voxels in a central region of interest. The gradient‐recalled echo signal equation is given by

(1) $$S=S_{0}\times \frac{1-e^{-\mathrm{TR}/T_{1}}}{1-e^{-\mathrm{TR}/T_{1}}\cos\theta }\text{sinθ}\times e^{-\mathrm{TE}/T_{2}^{*},},$$

where θ is the flip angle. Here, S_0_ incorporates both macroscopic magnetization (including density), signal detection (Faraday), and receive sensitivity. The measurements described previously thereby aimed at removing the T_1_ and T_2_* weightings of S_0_ to isolate its contribution alone. The SNR measurements performed at the three sites at 7 T were used as estimation of intersite variability,^25^, ^26^ which was then considered as uncertainty for the measurements at all field strengths. The SNR measurements were also corrected for the preamplifier SNR deterioration by multiplying their values by 10^NF/20^. The experimental data of SNR versus main field strength were then fitted with a power‐law dependence of the form SNR ∝ B_0_^n^. Combined with the uncertainty assessment described previously, a χ^2^ test was used to calculate goodness of fit and consistence between the data and model.^27^

### 2.5 Signal‐to‐noise ratio calculations

Hoult's intrinsic SNR formula^20^ for a spherical phantom of radius R and idealized spherical volume probe is given by

(2) $$\begin{array}{ll} \mathrm{d\Psi} & =M_{\mathrm{xy}}^{+}\mathrm{dV}\left(U_{0,0}-0.5U_{2,0}\right)\\ & \;\times {\left(\frac{12\text{πσκTΔν}}{kk^{*}}\int_{0}^{R}r^{2}j_{1}\left(\mathrm{kr}\right)j_{1}^{*}\left(\mathrm{kr}\right)\mathrm{dr}\right)}^{-1/2}, \end{array}$$

where dΨ is the intrinsic volume element FID SNR; k is the complex wavenumber inside the phantom; dV is the voxel volume; κ is Boltzmann's constant; T is the temperature; and Δν is the receiver bandwidth. The U_n,m_ are functions of the Bessel functions of the first kind and Legendre polynomials, which are constant at the origin (U_0,0_ = 1 and U_2,0_ = 0 at r = 0). The first term is the transverse magnetization, which grows linearly with field strength in the high temperature regime. The last term exhibits a more complex dependence on the electrical properties given that $k^{2}=\omega_{0}^{2}\mu_{r}\mu_{0}\varepsilon_{r}\varepsilon_{0}-i\omega_{0}\mu_{r}\mu_{0}\sigma$, which is in the argument of the spherical Bessel functions under the integral. In the low‐frequency regime, where kr < <1, one obtains j_1_(kr) ∼ kr/3 and an inverse square root dependence of the SNR on the conductivity. For high frequencies, this approximation does not hold and can lead to a faster than √σ change of SNR versus conductivity. Importantly, this formula does not depend on the receive B_1_^−^ field (it is present in the evaluation of the signal and noise, but cancels when taking their ratio). To a certain degree, it therefore appears reasonable to use a different but similar RF coil at 3 T, where receive sensitivity may differ slightly for another data point, as long as it is of a volume type. The relative gains of SNR with field strength obtained experimentally were compared with the ones predicted by (Equation 2) with the measured, yet almost constant, electric properties. Given the uncertainty of approximately 5% in the measurement of the electric properties, a robustness analysis of the results returned from the formula with respect to these parameters was performed with Monte Carlo tests by perturbing their values randomly and independently for the different field strengths to see their impact on the B_0_ exponent found after fitting.

Approaching the problem from the point of view of determining uiSNR, Lee et al^13^ demonstrated that only one divergence‐free mode contributes to the MR signal at the center of a sphere with uniform electrical properties, which led to the derivation of a precise analytical expression for the uiSNR in the center of a sphere. The returned expression is identical to the previous Hoult's result (Equations 48 and 6 in the two articles, respectively), thus proving that this SNR formula in fact can be considered the uiSNR at the center of a sphere of uniform properties.

## 3 RESULTS

**FIGURE 2:**
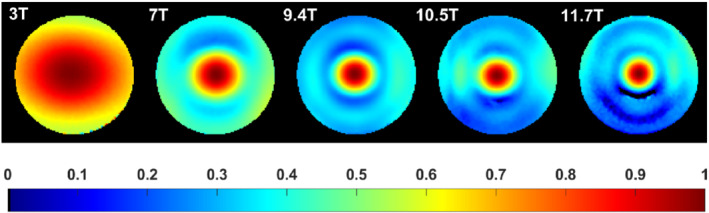
Measured B_1_^+^ field profile versus field strength (central axial view). The values are normalized with respect to the maximum at the center

The results of the AFI measurements normalized by the peak value at the center are represented in Figure 2. One can see the expected field focusing effect^20^, ^28^, ^29^ with increased RF field inhomogeneity versus field strength. At 10.5 T and 11.7 T, its extent was such that the accuracy of the measurement throughout the whole volume was impacted, given the dynamic range of detectable flip angles with the AFI sequence, making measurements in the periphery less accurate when targeting optimal sensitivity in the center with that sequence.

**FIGURE 3:**
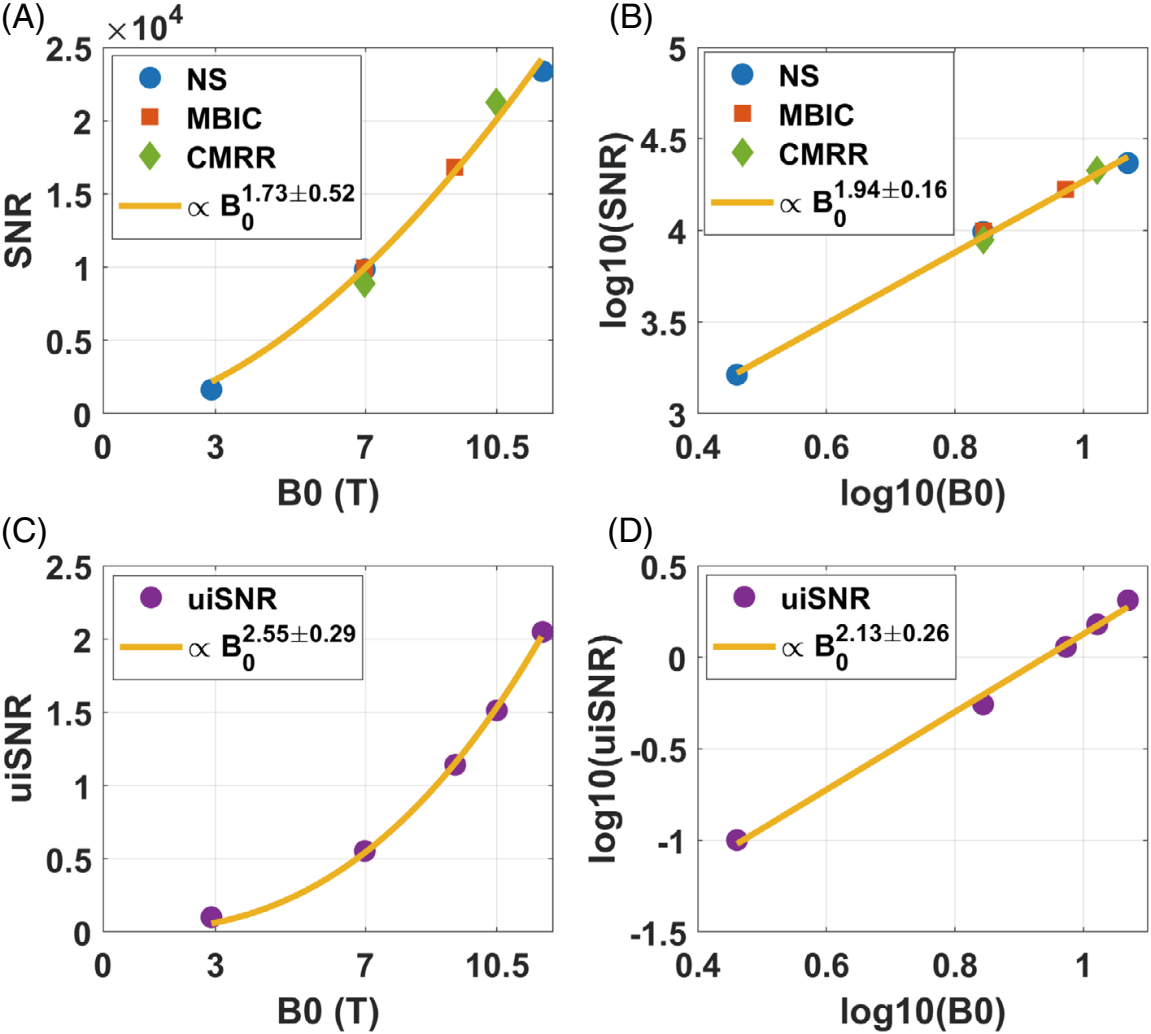
Measured SNR at the center of the phantom versus field strength in a multicentric study performed at Neurospin (NS), Maastricht University (MBIC), and University of Minnesota (CMRR) compared with ultimate intrinsic SNR (uiSNR) calculations. A, Untransformed data points with fit (raw SNR measurements, aside from flip angle, T_1_, T_2_*, and preamplifier NF corrections). B, Log–log representation of the same data with its linear fit. The 95% confidence intervals are indicated in the legends for the exponent of the fits. The UiSNR equivalent results are provided in (C) and (D)

Figure 3 reports the SNR measurement results versus field strength at the center of the sphere. Measurements performed at 7 T were within 1% agreement between NeuroSpin and MBIC and 10% with CMRR. The multiple measurements at 7 T obtained from the different sites were averaged before performing any analysis on the data. This intersite variability was then considered as uncertainty in the measurements at all field strengths to compute a goodness‐of‐fit measure.

Including preamplifier NF correction, a nonlinear fit to the data (Figure 3A) of the form aB_0_^n^ returned *n* = 1.73 ± 0.52 (95% confidence interval). A linear regression in the log–log domain returned *n* = 1.94 ± 0.16, while the theory^13^, ^20^ (Equation 2) returned 2.55 ± 0.29 and 2.13 ± 0.26 for the nonlinear and linear fits, respectively. Table 1 summarizes the results. Not compensating for the NF of the preamplifiers had only a small impact on the exponent found (1.72 vs 1.73 and 1.92 vs 1.94 for the nonlinear and linear fits, respectively). Given the confidence intervals, the exponents found with the experimental data with the nonlinear and linear fits are consistent (1.73 ± 0.52 vs 1.94 ± 0.16). The linear regression result with log–log data, however, is the one to be retained for several reasons. First, it yields a smaller uncertainty (± 0.16 vs ± 0.52). Second, a pure power‐law dependence over the entire 3T–11.7T range is not a priori expected; in fact, a more linear dependence on B_0_ can be expected at lower fields (see section 4). As such, the larger uncertainty arising from the fit on the untransformed versus the log–log transformed uiSNR data (± 0.29 vs ±0.26) also reflects the sensitivity as we deviate from a pure power‐law model. Finally, linear regressions in log–log domain are more appropriate when errors are multiplicative, such as uncertainties in preamplifier NFs or coil losses. The reduced χ^2^ returned a value of 0.76 (3 degrees of freedom), indicating good consistence between model and measurements given the estimated uncertainties.^27^

**TABLE 1:** Exponent results of the SNR = aB_0_^n^ fit for untransformed and log–log data

	Untransformed	log–log
uiSNR (measured electrical properties)	2.55 ± 0.29	2.13 ± 0.26
uiSNR (perturbed electrical properties) – Min and Max	2.10–3.21	2.06–2.21
Data	1.73 ± 0.52	1.94 ± 0.16

*Note*: The 95% confidence intervals are provided for the first and third lines. The Monte‐Carlo results (second line) are the min and max exponent values returned over 10 000 trials, where uiSNR calculations were performed with 0% to 5% random disturbance of the electrical property values, independently at the different field strengths.

**FIGURE 4:**
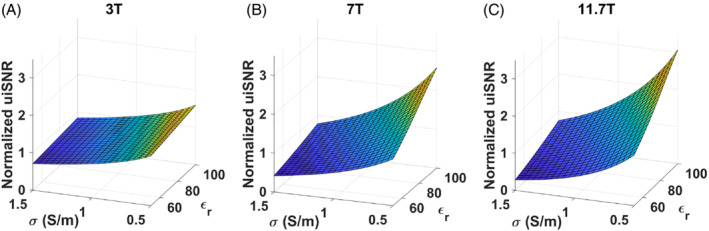
ultimate intrinsic SNR (uiSNR) versus electrical properties. The values are reported for the center of the sphere and are normalized with respect to the values found with the phantom properties (ε_r_ = 76 and σ = 1 S/m) at 3 T (A), 7 T (B) and 11.7 T (C)

Figure 4 illustrates surface plots of uiSNR with respect to ε_r_ and σ at 3 T, 7 T and 11.7 T, normalized to the value found in our phantom with ε_r_ = 76 and σ = 1 S/m, and obtained from Equation 2. It illustrates the deviations of the uiSNR results with respect to changes in electrical properties. At low fields, one recovers approximately a $1/\sqrt{\sigma }$ dependence of SNR. At high fields, SNR becomes more sensitive to changes of electrical properties and can particularly affect the results of the nonlinear fits. A Monte‐Carlo numerical test was performed with the uiSNR formula by disturbing the electrical property values randomly 10 000 times between 0% and 5% and independently for the different field strengths, repeating the uiSNR calculation of Equation 2 and the fits. The results are provided in the second row of Table 1 (min and max exponent values found over the repetitions), indicating again more robustness of the log–log fit. The surface plots of Figure 4 can convey how so few changes in the electrical properties can affect the B_0_ exponent: An increase (decrease) and decrease (increase), respectively, of σ and ε_r_ at low (high) fields, for instance, can pull the exponent up by increasing the gap between the SNR at low and high fields.

## 4 DISCUSSION AND CONCLUSIONS

Magnetic‐field strength dependence of SNR has proven to be a complex topic with a complex history. Early efforts undertaken subsequent to the introduction of MRI suggested SNR ∝ B_0_^1.75^ for nonconductive samples or very low frequencies (based on Faraday's law combined with the skin effect for increased resistance in the coil wires) where coil noise dominates, but more of a linear B_0_ dependence with conductive samples such as human tissue.^30^, ^31^ This prediction has dominated the thinking on the topic for a long time, even though these were near‐field approximations that did not consider wave propagation and consequent phase variation over the sample at or near far‐field conditions. Such wave effects, however, started becoming clearly noticeable at ultrahigh magnetic fields of 4 T and 7 T, where human head images showed highly nonuniform excitation and SNR when using volume transmit/receive coils.^29^, ^32^ Subsequent experiments at 7 T clearly ascribed the source of this inhomogeneity to the presence of the wave effects and the consequent spatial variation in the phase and amplitude of the transmit and receive B_1_ (B_1_^+^ and B_1_^−^, respectively) determined by a given sample‐coil configuration.^1^, ^28^, ^33^ These early studies also underlined the difficulty of reaching conclusions on the field dependence of SNR from experiments due to the presence of the aforementioned spatial nonuniformities and the role played by the RF coil layout in defining them.^28^ Such experimental complications are avoided in analytical calculations^18^, ^20^ or electromagnetic simulations of the field dependence of the uiSNR, which is independent on the coil configuration and predicts supralinear gains in SNR with B_0_ centrally and approximately linear gains in the periphery in spherical phantoms mimicking the electrical properties of human tissue^13^, ^15^ or realistic human head models.^14^

Experimental data indicating supralinear B_0_ dependent SNR gains were published by Pohmann et al,^21^ who reported SNR ∝ B_0_^1.65^ in the human head from data obtained at 3 T, 7 T, and 9.4 T using 32‐channel receive array coils. Unlike the uiSNR calculations, however, the SNR gains observed by Pohmann et al were relatively uniform over the entire head, although a slightly higher gain was apparent centrally, especially when comparing 3 T and 7 T. The different RF coils used in that study is a confound that can potentially account for this observation. Our 3T coil, although it was different from the other coils at higher field strengths, was unshielded and therefore probably led to a more optimal scenario at 3 T than with the shielded case.^22^, ^23^ Although Hoult's theory does not predict any SNR change with the diameter of its idealized and lossless spherical probe, we cannot rule out in practice a volume coil design with different dimensions and more optimal SNR. As a result, interpretation of the presented results depends on the validity of the coil near‐optimality assumption at the center of the phantom.

In this work we focused on a set of measurements using a well‐defined geometry and region (center of a sphere) amenable to analytical calculations,^13^, ^20^ thereby making the comparison between measurements and theory ideal. We have also used the same coil design from 7T to 11.7T field strengths and a similar coil at 3 T, nearly identical setups including also phantom, positioning, temperature, electrical properties, and examined five different magnetic field strengths covering a wider range (3 T–11.7 T) compared with the Pohmann et al study.^21^ The coil selected in our case, a birdcage coil available at all sites, is ideal for SNR measurements at the center of a spherical sample but not in the periphery^18^; in the center of a sphere, B_1_^+^ and B_1_^−^ for such a volume coil always adds constructively, regardless of B_0_ magnitude, whereas in the periphery both B_1_^+^ and B_1_^−^ are diminished due to destructive interferences,^28^ as observed in our experimental data (Figure 2). Consistency between the setups could be confirmed by measurements on three different 7T MRI machines located at the three different sites, yet revealing intersite variability that could be attributed to slightly different coil losses or phantom changes. The methodology and data thereby allow extracting more easily the contribution of the main magnetic field alone on SNR, returning a power‐law dependence of B_0_^1.94 ± 0.16^, with a χ^2^/dof = 0.76 (3 degrees of freedom), given the estimated uncertainty. The gain found experimentally thus was smaller than the 2.13 exponent derived from uiSNR theory. Possibilities to explain the deviation between measurements and theory is decreased skin depth on the conductors of the coil and consequently more resistance as well as more radiation losses with increasing magnetic field strength. Imperfections of the phantom (shape and uniformity of the properties) and uncertainties in the electrical property values could also play a role (see Table 1). Simulations of uiSNR (34) and experiments in the human brain (35, 36) with multichannel receive arrays demonstrate little SNR improvement in unaccelerated imaging for greater than ∼32 channels at the center. Therefore, with the agreement between Hoult's idealized CP‐volume coil SNR result (18, 20) and Lee's uiSNR derivation (13, 15), it can be reasonably argued that B_0_ dependence of central SNR we report can be extrapolated to multi‐receive arrays in the absence of acceleration. Hence our results can be compared to those reported by Pohmann et al (21) where a 32 channel receive array was employed for the measurements. Our exponent of 1.94 agrees reasonably well with the value of 1.65 reported by them. In addition to a larger sample volume in vivo (larger filling factor), it is also worth stressing that our study dealt with a phantom of nearly constant electrical properties versus frequency while the in‐vivo data reported in (21) naturally incorporated the variations of electrical properties of biological tissues with field strength. The difference between the exponents therefore can also be a consequence of those changes. It is also worth pointing out that Hoult's and uiSNR theory (Equation 2) do not predict a pure B_0_^n^ dependence of the SNR versus field strength, but more of a linear followed by a supra‐linear regime at low and high field respectively. Just as in (21), fitting our experimental data with the formula above as a result was purely of practical interest to convey a simple law, as long as the fits were of good quality.

The approximately quadratic dependence on B_0_ reported in this study as well as in theoretical calculations at first may seem contradictory to the concept that noise with conductive samples is dominated by the sample. After all, it is that particular assumption that led to predictions of SNR ∼ B_0_ in early considerations.^30^, ^31^ However, at the frequencies involved at ultrahigh magnetic fields, the wave behavior and the resulting spatial variation in phase over the sample essentially leads to averaging of the electric fields. This averaging in effect reduces the “sample noise” in the measurement. The same would be observed for MR detected signals, if it was not for the fact that those signals are spatially encoded and the relevant variation in phase are those over a voxel. Without the spatial encoding, however, the FID from the sample would also be attenuated.^20^

To conclude, we report SNR measurements at the center of a sphere of known electrical properties from 3 T to 11.7 T and with nearly identical experimental conditions to attempt at isolating the SNR gain from the main magnetic field alone. The dependence of the SNR with field strength is found experimentally to be proportional to B_0_^1.94^ and is reasonably close to the behavior predicted by uiSNR theory.

## FUNDING INFORMATION

European Union Horizon 2020 Research and Innovation program (885876; AROMA); a French government grant managed by the Agence Nationale de la Recherche under the program “Investissements d'avenir” (ANR‐21‐ESRE‐0006); and the National Institutes of Health (U01 EB025144 and P41 EB027061)
